# Supplementary material for: Regional variation in fire weather controls the reported occurrence of Scottish wildfires
Source: PeerJ. 2016 Nov 2;4:e2649. doi: 10.7717/peerj.2649 (PMC5101601; doi:10.7717/peerj.2649)
Supplement: Supplemental Information 1 — The effect of data set size and fire occurrence frequency on the results of Thiel-Sen analysis as described by Eastaugh, Arpaci & Vacik (2012) [file peerj-04-2649-s001.docx]

REGIONAL VARIATION IN FIRE WEATHER CONTROLS THE REPORTED OCCURRENCE OF SCOTTISH WILDFIRES

*SUPPLEMENTARY DOCUMENT – APPENDIX I*

**The effect of data set size and fire occurrence frequency on the results of Thiel-Sen analysis as described by Eastaugh et al. (2012)**

Eastaugh et al. (2012) claim a perfect discriminator would have a slope approaching zero and an intercept approaching 100. They state smaller slopes and larger intercepts indicate better index performance. However, the results of their method are sensitive to the overall size of the dataset, the associated number of fires recorded and the distribution of index values across the days in the dataset. In particular intercept is sensitive to the number of fire days and slope is sensitive to the number of days in the dataset. Caution must be used when using the method to compare between different datasets. This is demonstrated in the examples below. Thiel-Sen regression analyses were completed using the “mblm” package (Komsta 2015) in R 3.1.2 (R Core Team 2014). An R script for this sensitivity analysis is provided with this paper.

- **Example 1**: Dataset of 100 days with three fires. The hypothetical index is a linear perfect predictor (range 1-100) over the range of the data. Fires occur on percentiles 98, 99 and 100.
  - *Results: Intercept = 97, Slope = 1*
- **Example 2**: Dataset of 100 days with fifty fires. The hypothetical index is a linear perfect predictor (range 1-100) over the range of the data. Fires occur on percentiles 50-100.
  - *Results: Intercept = 50, Slope = 1*
- **Example 3**: Dataset of 365 days with fifty fires. The hypothetical index is a linear perfect predictor (range 1-100) over the range of the data. Fires occur on percentiles 50-100.
  - *Results: Intercept = 86.30, Slope = 0.27*
- **Example 4**: Dataset of 365 days with ten fires. The hypothetical index is calculated as per equation 5 in Eastaugh et al. The index is a perfect predictor and fires occur on percentiles 97.26 – 100.00.


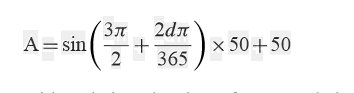


- - *Results: Intercept = 96.99, Slope = 0.27*
- **Example 5**: Data set of 365 days with fifty fires. The hypothetical index is calculated as per equation 5 in Eastaugh et al. The index is a perfect predictor and fires occur on percentiles 86.58 – 100.00.
  - *Results: Intercept = 86.30, Slope = 0.27*
