# Supplementary material for: Regional variation in fire weather controls the reported occurrence of Scottish wildfires
Source: PeerJ. 2016 Nov 2;4:e2649. doi: 10.7717/peerj.2649 (PMC5101601; doi:10.7717/peerj.2649)
Supplement: Supplemental Information 2 — Supplementary tables showing summaries of generalized model fits relating fire weather characteristics to wildfire occurrence. Supplementary Figure showing the distribution of large and all wildfires across in relation to season and fire weather [file peerj-04-2649-s002.docx]

REGIONAL VARIATION IN FIRE WEATHER CONTROLS THE REPORTED OCCURRENCE OF SCOTTISH WILDFIRES

*SUPPLEMENTARY TABLES AND FIGURES – APPENDIX II*

**Supplementary Tables: Summary of generalized model fits relating fire weather characteristics to wildfire occurrence**

**Table S1: Generalized linear model of wildfire occurrence as a function of FFMC**

Model structure

Fire ~ FFMC + Urban + season + Region + FFMC:Urban + FFMC:season + FFMC:Region

**Estimate Std. Error z value Pr(>|z|)**

*(Intercept) -3.085766 0.434572 -7.101 1.24e-12 ****

FFMC 0.047550 0.006231 7.632 2.32e-14 ***

Urban.Urban 0.801428 0.271040 2.957 0.003108 **

season.spr -1.287787 0.365971 -3.519 0.000433 ***

season.sum -0.544319 0.430081 -1.266 0.205649

season.win 0.927454 0.427944 2.167 0.030217 *

Region.Grampian -2.303690 0.517463 -4.452 8.51e-06 ***

Region.Highlands -0.842934 0.378915 -2.225 0.026108 *

Region.Lothian -0.509665 0.421253 -1.210 0.226325

FFMC:Urban.Urban -0.010662 0.003780 -2.820 0.004799 **

FFMC:season.spr 0.018966 0.005236 3.622 0.000292 ***

FFMC:season.sum 0.001533 0.005893 0.260 0.794692

FFMC:season.win -0.009848 0.006482 -1.519 0.128681

FFMC:Region.Grampian 0.027773 0.007063 3.932 8.42e-05 ***

FFMC:Region.Highlands 0.012169 0.005387 2.259 0.023869 *

FFMC:Region.Lothian 0.004954 0.005936 0.834 0.404015

**Table S2: Generalized linear model of wildfire occurrence as a function of ISI**

Model structure

Fire ~ ISI + Urban + season + Region + ISI:Urban + ISI:season + ISI:Region

**Estimate Std. Error z value Pr(>|z|)**

*(Intercept) -1.11421 0.15931 -6.994 2.67e-12 ****

ISI 1.29340 0.14212 9.100 < 2e-16 ***

UrbanUrban 0.19343 0.09090 2.128 0.033344 *

seasonspr -0.41390 0.12372 -3.345 0.000822 ***

seasonsum 0.06518 0.13020 0.501 0.616635

seasonwin -0.10864 0.16879 -0.644 0.519834

RegionGrampian -0.39454 0.16842 -2.343 0.019148 *

RegionHighlands 0.07863 0.14118 0.557 0.577557

RegionLothian 0.16221 0.15346 1.057 0.290495

ISI:UrbanUrban -0.13630 0.07306 -1.866 0.062100 .

ISI:seasonspr 0.38677 0.11509 3.361 0.000778 ***

ISI:seasonsum -0.43787 0.10850 -4.036 5.44e-05 ***

ISI:seasonwin 0.63830 0.20520 3.111 0.001867 **

ISI:RegionGrampian 0.04249 0.13231 0.321 0.748104

ISI:RegionHighlands -0.03046 0.12256 -0.249 0.803697

ISI:RegionLothian -0.26742 0.12745 -2.098 0.035878 *

**Table S3: Modeled probability of an occurring fire being of high magnitude as a function of FWI**

Model structure

Large ~ FFMC + Urban + Region + season + season:FFMC

**Estimate Std. Error z value Pr(>|z|)**

*(Intercept) -4.84507 0.99752 -4.857 1.19e-06 ****

FFMC 0.01485 0.01252 1.187 0.2353

UrbanUrban -0.93003 0.15179 -6.127 8.94e-10 ***

RegionGrampian 2.46416 0.33619 7.330 2.31e-13 ***

RegionHighlands 1.82768 0.33007 5.537 3.07e-08 ***

RegionLothian 0.12702 0.39587 0.321 0.7483

seasonspr -1.76767 1.21255 -1.458 0.1449

seasonsum -0.95234 1.42317 -0.669 0.5034

seasonwin -3.35086 1.94198 -1.725 0.0844 .

FFMC:seasonspr 0.02648 0.01571 1.685 0.0919 .

FFMC:seasonsum 0.01178 0.01817 0.649 0.5166

FFMC:seasonwin 0.04937 0.02528 1.953 0.0509 .

**Supplementary Figure: The location of former regional Fire Authorities in Scotland**


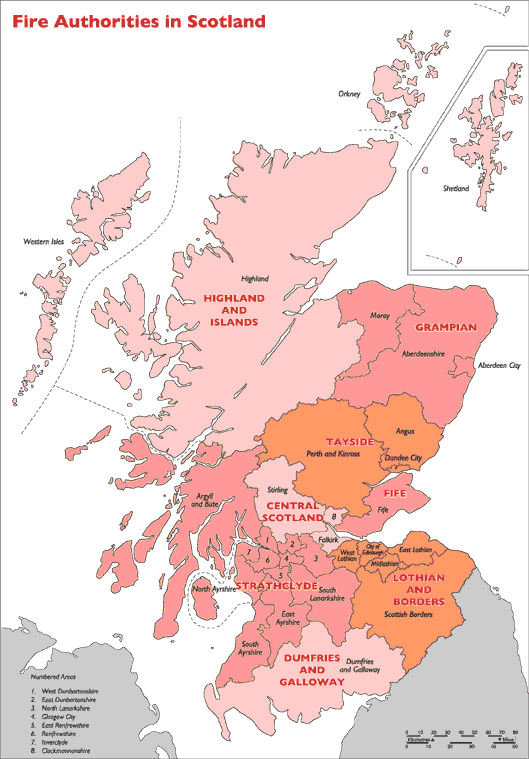


**Figure S1:** The location of former regional Fire Authorities in Scotland. Our project utilized data provided by the Dumfries and Galloway, Grampian, Highlands and Islands and Lothian and Borders regions. Regional Fire Authorities in Scotland have since been merged into a single Scottish Fire and Rescue Service.

The map is reproduced from: <http://www.gov.scot/Publications/2005/10/2592612/26126> [accessed 07-Sep-2016].

**Supplementary Figure: Distribution of large and all wildfires across in relation to season and fire weather**

**Figure S2**: The distribution of all wildfires (crosses) and large fires (black circles) from our 4.5 year data set across seasons and fire day FFMC values. Seasons are shown as different colours: winter = grey; spring = green; summer = dark green; autumn = blue. Days between the two red lines are outwith the legal managed burning season (October 1^st^ – April 15^th^)
